# Is forest bathing a panacea for mental health problems? A narrative review

**DOI:** 10.3389/fpubh.2025.1454992

**Published:** 2025-02-20

**Authors:** Hongxia Chen, Zhongji Meng, Jie Luo

**Affiliations:** ^1^Institute of Biomedical Research, Department of Infectious Diseases, Regulatory Mechanism and Targeted Therapy for Liver Cancer Shiyan Key Laboratory, Hubei Provincial Clinical Research Center for Precise Diagnosis and Treatment of Liver Cancer, Taihe Hospital, Hubei University of Medicine, Shiyan, Hubei, China; ^2^School of Public Health, Hubei University of Medicine, Shiyan, China; ^3^Hubei Key Laboratory of Embryonic Stem Cell Research, Taihe Hospital, Hubei University of Medicine, Shiyan, Hubei, China; ^4^Department of Infectious Disease, Taihe Hospital, Hubei University of Medicine, Shiyan, Hubei, China; ^5^Hospital Affairs Office, Taihe Hospital, Hubei University of Medicine, Shiyan, Hubei, China

**Keywords:** forest bathing, pressure, anxiety, depression, cortisol, mental health

## Abstract

**Background:**

The fast pace of modem life brings great pressure, which lead to physical and mental health issues. Researches have demonstrated that forest bathing can considerably alleviate symptoms of depression and anxiety, eliminate negative emotions and promote mental wellbeing. We presented evidences of the positive impact of forest bathing on mental health in the context rapid urbanization and surging health needs in the post-pandemic era, and outlined the current insights into the related factors affecting the effect of forest bathing, as to provide directions for future interventions or research.

**Method:**

The electronic databases PubMed, Cochrane Library, Embase, Web of Science Core Collections and the China Academic Journals (CAJ) offered through the Full-text Database (CNKI) were searched for relevant studies published from the inception of the databases to December 2024. The initial search strategy was performed using keywords, MeSH terms, and free text words such as “forest bathing”, “forest medicine”, “mental health pressure”, “anxiety”, “depression”, “cortisol”, etc.

**Results:**

The synthesis of the findings in the included studies revealed that forest bathing interventions might improve mental and physical health, reduce blood pressure, improve sleep quality and boost immunity, as well as alleviate depression, anxiety, and stress. Furthermore, the effect of forest bathing on mental health indicators and the differences in these results among different populations varied. Forest environment, tree species, exposure duration, season, composition and concentration of volatile organic compounds have an impact on the effect of forest bathing.

**Conclusions:**

Forest bathing were effective in lowering cortisol levels, reducing sympathetic nerve activity, as well as improving negative mood, which could serve as a non-pharmacological treatment for mental health in the general population.

## 1 Introduction

With the development of society and economy, the process of urbanization is accelerating rapidly. Fast- rhythm urban life and environmental pollution have increased people's life pressure, leading to a series of physical and mental health issues. Additionally, the COVID-19 pandemic in 2019 has resulted in strict quarantine policies, exacerbating existing mental health problems. Studies have shown that quarantine has many negative psychological effects that might persist a long time after quarantine orders were lifted (1). Depression is the primary cause of disability worldwide and a significant contributor to the global burden of disease, and the prevalence of depression varies from 2.2%–26.8% in the general population (2). In addition, the prevalence of clinically relevant depressive symptoms among COVID-19 survivors was found to range from 21% to 45% (3). Chen et al. (4) investigated the changes in the prevalence of depression and anxiety among Chinese adolescents during the COVID-19 epidemic and found that it increased significantly after the outbreak compared with the data reported during the COVID-19 epidemic. In the initial survey, the prevalence of depression and anxiety was 36.6% and 19%, respectively. In the second survey, the prevalence of depression and anxiety increased rapidly to 57.0% and 36.7%, respectively.

The forest environment has been favored by people for its peaceful atmosphere, beautiful scenery, mild climate, pleasant aroma and fresh air for a long time. Exposure to forest environments promotes human health, with amounting relevant studies in this area increased rapidly (5). Scientists in Japan have explored the role of forests in preventing non-communicable diseases and have come up with a new concept called “forest bathing” (Shinrin-Yoku in Japanese) (6). Forest bathing is a nature-based therapy that directs the participant's attention toward their sensory experience, encouraging exploration of the surrounding forest through sight, hearing, touch, smell and taste (7). Forest bathing has been proved to improve mental and physical health, not only by lowering blood pressure, improving sleep and boosting immunity, but also by improving depression, anxiety and stress. Japanese scholars have done a lot of related research on the health promotion of forest bathing, and have developed a new science “forest medicine” (8). Undoubtedly, Japanese boasts the world's highest life expectancy, which was attributed in part to the popularity of forest bathing in Japan.

Currently, forest bathing has become a global trend as a natural response to the fast-paced and busy daily life of modern society. It is noteworthy that Clifford Amos, being considered as one of the pioneers of introducing the practice of forest bathing to the West, presented practical guidance and the philosophy of Shinrin-yoku, the Japanese art of immersing oneself in nature in the books named by “Your Guide to Forest Bathing: Experience the Healing Power of Nature” and “Guide's Handbook of Forest Therapy” (9). Studies have shown that it can increase parasympathetic activity, reduce sympathetic activity, and promote relaxation and psychological sedation (10). Therefore, this review illustrated the positive effects of forest bathing on mental health in the context of urbanization and the post-pandemic era, and explored the related factors affecting the effect of forest bathing. It highlighted the potential therapeutic role of forests in mental health, provided evidence-based support for forest conservation, and offered a scientific basis for the effective development of forest healing programs for the benefit of the general population, especially for older adults.

## 2 The way of forest bathing and the appropriate crowd

Individual demand for forest therapies varies, with older people showing greater enthusiasm than younger people in terms of their preference for forest therapies. In forest therapy in form of mountain climbing or hiking, the physical burden on the older adult and women is significant, and additional safety management is required to prevent potential injuries. Therefore, if participants have sex- or age-specific diseases, the content of forest therapy program should be personalized for their age and gender to ensure safety.

### 2.1 Meditation

Cognitive impairment can occur in the older adult population or patients with depression and anxiety due to physiological and pathological factors. At the same time, due to the influence of physical fitness, physical condition and other related factors, older adults and people with mobility problems might choose to meditate in the forest instead of other forest projects. As we known, meditation is an ancient mindfulness practice with roots in Buddhist and Hindu cultures (11). Mindfulness meditation, a meditation tradition originally derived from Buddhism, has been applied to a variety of physical and mental health conditions and has received considerable attention in psychological research (12). A growing body of research on the behavioral and neurophysiological effects of meditation has proved that meditation can be used to improve mood, reduce stress, and affect various cognitive functions in both normal and patient populations (13, 14). Basso et al. found that daily brief meditation has benefits not only in reducing negative emotional states, but also relieving stress, as well as improving attention and memory (14). Active meditation in the forest can be done while listening to the sound of wind, water and bird singing. In particular, meditation in the forest is about feeling and understanding the life force of nature through communication with nature. The effects of meditation in the forest are enhanced by forest healing factors, such as the sound of water in the valley and the scent of pine trees, which related to the eco-psychology. In addition, forest trees are able to release specific volatile organic compounds, which show anti-inflammatory and antioxidant effects on the respiratory tract and general relaxing, calming and anti-anxiety effects on the central nervous system after inhalation and systemic absorption. Overall, forest meditation is a perfect program for people with mobility issues.

### 2.2 Climbing and hiking

Walking is a common physical activity that plays an important role in disease prevention. Research has shown that hiking in forests promotes health and helps to minimize psychological stress in anxious individuals (15). Moreover, forest hiking can provide positive mental benefits for those with severe depressive symptoms. In addition to forest hiking, mountain climbing offers a range of benefits. Fast-tempo society brings great pressure to people, which is easy to cause anxiety, irritability, depression and other negative emotions, and we need to find a suitable way to release it. Climbing mountains can help relieve negative emotions, psychological stress, and help people embrace life with a more positive attitude. Jia et al. conducted a 3-day, 1.5-h per day walking test in both forest and city environments (16). The results showed that walking in the forest significantly reduced cortisol and adrenaline levels when comparing to walking in the city. Additionally, aerobic exercise in a forest environment with a high concentration of negative oxygen ions can improve blood circulation to the bones and enhance cardiopulmonary function. The forest's negative oxygen ions can enhance the ventilation function of the lung, regulate nerves, stimulate the spirit, improve sleep quality, reduce blood pressure and promote relaxation. These effects include calming, sedation, blood pressure reduction and fatigue elimination.

### 2.3 Yoga in the forest

Yoga is an ancient traditional form of exercise that originated in India and is based on a holistic health system that integrates mind, body and spirit, which is considered a form of complementary and alternative medicine for developing and maintaining good physical and mental health. Practicing yoga can help train the brain, improve thinking patterns and enhance the ability to cope with stress. Research has found that yoga can reduce cortisol levels by downregulating the hypothalamic-pituitary-adrenal axis (17). Two case-control studies have confirmed that practicing yoga can effectively alleviate and prevent the occurrence of perinatal anxiety and depression by significantly reducing cortisol levels. In both studies, short yoga practices consisting of postures, stretches, respiratory conditioning and relaxation techniques resulted in decreased cortisol levels (18). Thirthalli et al. (19) revealed that patients with major depression had significantly lower cortisol levels after 12 weeks of regular yoga practice compared to patients who only received antidepressants. One of the main mechanisms by which Yoga improves mood by increasing levels of gamma-aminobutyric acid (GABA) in the brain (20), while there was a correlation between increased levels of thalamus GABA and improved mood as well as decreased anxiety after practicing yoga (21). The forest offers plenty of elements that contribute to a sense of comfort, including beautiful scenery, fresh air, pleasant birdsong, plant antibacterial agents and negative oxygen ions. In conclusion, practicing yoga in the forest is a whole-hearted exercise and relaxation, which can not only relieve and prevent anxiety and depression, but also take advantage of the various environmental factors of the forest to synergistically promote overall human health.

### 2.4 Mindfulness

Mindfulness is defined as the inherent capacity for meta-awareness, enabling individuals to focus on present-moment experiences while avoiding cognitive biases. This ability can be cultivated through simple meditative attentional practices and structured training programs such as mindfulness-based interventions (MBIs). Research (22) has demonstrated that mindfulness training can effectively reduce stress, anxiety, and burnout, while promoting an enhanced quality of life across diverse settings. Mindfulness and Shinrin-yoku (SY), commonly referred to as forest bathing, have demonstrated potential efficacy in alleviating mental health issues associated with the COVID-19 pandemic and other uncertain events. Studies have shown a significant positive correlation between exposure to nature, mindfulness practices, and improved psychological wellbeing. During periods of uncertainty, such as during the COVID-19 pandemic, integrating mindfulness with SY might be particularly beneficial for at-risk groups, including individuals experiencing depression, loneliness, and social isolation, as well as specific populations such as college students, veterans, and professionals under high stress (23). A pilot intervention study (24) demonstrated the trends that forest bathing could improve medical residents' psychological wellbeing and mindfulness, which might be a valuable clue for the improvement of mental and physical health among the healthcare providers. In the study investigating the impact of the WAYA wilderness programme (25) (which included activities such as hiking, backpacking, kayaking, rock climbing, bushcraft, and mindfulness) on the health of childhood, adolescent, and young-adult (AYA) cancer survivors, it was found that participants experienced enhanced trust and self-confidence, personal growth, relaxation, and stress recovery when immersed in nature. Nature provided a supportive environment that facilitated respite from daily challenges and stimuli, thereby fostering a deeper connection with the natural world.

### 2.5 Forest therapy

There is another forest bathing in the European form, which refers to a series of structured activities and mindfulness interventions that utilize the forest environment in various ways to promote health. These are sessions led by a forest bathing guide (forest therapy provider) that include elements of mindfulness in nature and specific activities engaging the senses of hearing, smell, and touch (26). They are based on structured tasks (invitations) performed by participants in the guide's presence. These activities not only involve engaging all the senses but also stimulate imagination and explore how elements of nature reflect the laws governing life, incorporating mindfulness or even elements of cognitive behavioral therapy (CBT). In addition, Zhang et al. (27) conducted a systematic review, distinguished between forest therapy programs, forest exposure, forest exercise, and forest therapy, and gave a relatively precise definition of a forest therapy program, which will be the main direction of our future research efforts, aiming to enhance the health effects of forests for the benefit of the general public.

## 3 The assessment methods and biomarkers of mental health in forest bathing interventions

### 3.1 Cortisol

Cortisol is commonly used as a biological indicator of stress and a dominant measure in studies assessing the therapeutic effects of forest bathing. It was well-known that cortisol plays an important role in regulating many basic biological systems in the human body, such as metabolic, immune, inflammatory processes, and disruption of its circadian rhythm may affect the function of these systems and may have health implications over time (28). Cortisol, a glucocorticoid released by the adrenal gland, is a sensitive and reliable stress biomarker, and salivary cortisol levels reflect free serum cortisol concentrations. Stress stimuli activate the pituitary-adrenal axis, leading to increased cortisol production and having the potential to disrupt the circadian release of this hormone. The energy and biological substrates necessary to cope with stressful stimuli or escape threats. Although cortisol secretion is an adaptive short-term response to stress, chronic stress-related cortisol secretion causes the cortisol-producing system, the hypothalamic-pituitary-adrenal (HPA) axis, to become dysregulated (29). This means that cortisol secretion does not follow the expected secretion pattern. While stress-relieving methods, such as forest bathing, are thought to help improve stress resilience, which significantly reduce cortisol levels in the short term, and may improve cortisol arousal responses in the long term (30).

### 3.2 Serotonin

Serotonin is a neurotransmitter that affects movement, emotion regulation and sleep (31). A lack of serotonin increases depression, anxiety (32) and impulsivity (31), while adequate serotonin contribute to feelings of comfort, pleasure and happiness (33). It was reported that patients with major depression had lower serotonin levels in the serum (34, 35). Moroianu et al. found that serum serotonin concentration was inversely associated with depression scores in patients with type 2 diabetes who presented with anxiety and depression (36). Forest bathing may have a potential preventive effect on depression, which mainly showed that Shinrin-Yoku significantly increased serum serotonin levels, improved POMS activity scores and reduced fatigue scores (37). Park et al. found that meditation-oriented forest therapy was able to increase serotonin levels, which helped prevent disease and improve quality of life (38).

### 3.3 Blood pressure and heart rate variability

Research has suggested direct and independent associations between anxiety severity, resting blood pressure and muscle sympathetic nerve activity in healthy adults without comorbid cardiovascular or metabolic disease, which indicated that anxiety might play an important role in the regulation of sympathetic control and resting cardiovascular function (39). Blood pressure is regulated by both sympathetic and parasympathetic nervous systems. Specifically, sympathetic activity increases and parasympathetic activity decreases blood pressure, and there is a significant correlation between blood pressure and urinary epinephrine and norepinephrine (40). This suggests that forest therapy might reduce systolic and diastolic blood pressure, urinary epinephrine and serum cortisol in middle-aged men with elevated blood pressure by reducing sympathetic activity, thereby preventing the progression of hypertension and reducing the risk of cardiovascular and renal disease (41). It was well-known that blood pressure and the level of heart rate variability (HRV) influence each other, and that diastolic blood pressure is more closely related to the autonomic nervous system than systolic blood pressure (42). The forest environment was found to significantly lower mean systolic blood pressure, while also significantly enhancing the mean power of the high-frequency component of HRV related to parasympathetic activity when the subjects felt relaxed while immersed in the forest (43).

### 3.4 The profile of mood states (POMS)

The profile of mood states (POMS) provides a rapid and effective method to assess transient, fluctuating active mood states, which is widely used in mood and mental health assessment and is considered to have good internal validity (44, 45). POMS can measure the following six emotional states: Tension-Anxiety (T-A), Depression-Dejection (D), Anger-Hostility (A-H), Vigor-Activity (V), Fatigue-Inertia (F) and Confusion-Bewilderment (C). Studies showed that forest bathing significantly increased vitality scores and decreased fatigue scores on the POMS test (39). The finding confirmed that forest bathing can relieve people's psychological tension, depression, anger, fatigue and confusion, and can enhance people's psychological vitality (37).

## 4 The effects and mechanisms of forest bathing on mental health

Forest bathing has been found to have several benefits, including reducing stress hormones such as epinephrine and norepinephrine in urine, decreasing sympathetic nerve activity, and relieving negative emotions such as nervousness, anxiety, anger, depression, fatigue and confusion in the POMS test. Furthermore, forest bathing increases vitality and parasympathetic nerve activity in men and women (42–47). Currently, some scholars have conducted research to explore the impact of forest bathing on mental health indicators and the differences in these results among different populations, and found that total mood disturbance (TMD) decreased after forest-based health and wellness (FHW) by 38.8 points on average, and the decompression effect of FHW experience showed some variability among individuals. More specifically, there were gender differences in alleviation of fatigue and puzzlement with greater impact for females than males (48). In the field study (49) to explore and compare the effects of a nature-based and an exercise-based social prescribing scheme on mental wellbeing and sleep in a primary care population, the result showed that perceived mental health improvement only was observed in the Nature-group, as well as better sleep quality. Meanwhile, this study indicated that nature-based interventions are beneficial for those in poor health.

### 4.1 Stress and job burnout

In modern urban societies, chronic stress and the inability to release stress is recognized as a growing problem with long-term health implications. Stress can be caused by emergencies, family stress, work pressure, or both, which might be a major challenge to personal health and have an adverse impact on people's work and daily life. Workplace stress has become a growing concern for organizations. Job burnout has attracted more and more attention in the society, which is a common syndrome in the social population, and its symptoms include being down in spirits and low sense of personal achievement (50). Kavanaugh et al. conducted a study to evaluate the impact of participating in a forest bathing intervention on the level of burnout among physicians and other medical staff. The results showed that forest bathing can reduce burnout symptoms, and the intervention group had a significant decrease in burnout scores when comparing to the control group after the test (51). In addition, a systematic review and meta-analysis by Antonelli et al. found that compared with the control group, the cortisol level of the intervention group in the forest group was significantly reduced after the intervention, which revealed that forest bathing can significantly affect cortisol levels in the short term, thereby reducing stress (30). The meta-analysis by Stalder et al. (52) reported that there was a positive associations of hair cortisol concentrations (HCC) with stress-related anthropometric (body mass index, waist-to-hip ratio) and hemodynamic measures (systolic blood pressure), which revealed a potential mechanism between chronic stress exposure and mental health.

### 4.2 Anxiety and depression

Anxiety and depression are among the most common psychological diseases in society. Patients with depression usually have the characteristics of anxiety, and vice versa, and both disorders may occur simultaneously. According to the World Health Organization's World Mental Health Survey, about 10% of people with mild depressive symptoms will develop to clinical depression (53). Furuyashiki et al. (54) explored the physical and psychological effects of a day of forest bathing on workers, and found that among 155 workers, 37% showed a tendency to depression, with the highest rates among the young and middle-aged. After forest bathing, all participants had significant decreases in SBP, DBP, and POMS negative items. Particularly, depression-prone individuals showing greater improvement on many POMS items than non-depression-prone individuals, however, many items no longer differed between those with and without depression. Li et al. (37) found that forest bathing reduced urinary stress hormones such as epinephrine and norepinephrine, alleviated sympathetic activity and negative emotions such as nervousness, anxiety, anger, depression, fatigue and confusion in the POMS test, and enhanced the sense of vitality and parasympathetic activity in men and women, showing a relaxation effect. Furthermore, it was found that forest bathing had a significant positive effect on the sleep quality of patients with depression. Most patients with depression have sleep disorders, which is a common and key symptom affecting most patients with depression. These findings suggest a potential preventive effect of forest bathing on depression (or a depressive state). In addition, Chun et al. found that forest therapy based on eco-psychiatry can effectively relieve depression and anxiety in patients with chronic stroke, and can improve antioxidant capacity in stroke patients, which might be a valuable intervention for patients who cannot be treated with standard therapies (55). The systematic review by Lee et al. found that although all included studies varied in terms of sample characteristics and intervention types, overall, these studies demonstrated that forest therapy was effective in improving depression, especially for the adults with mental health issues (56).

### 4.3 Cognitive impairment and mental disease

Forest bathing is thought to have therapeutic effects on neurodegenerative diseases, reducing heart rate and blood pressure and increasing parasympathetic activity in patients (57). In addition, forest exposure significantly reduced the levels of interleukin-6 and glutathione peroxidase, which are involved in defending against pathogens and reducing oxidative stress, respectively (58). Forest bathing has been shown to reduce malondialdehyde levels, while elevated levels of malondialdehyde are considered to be one of the manifestations of oxidative stress (59). These positive effects are partly due to terpenes, which are emitted into the atmosphere by trees and other plants. Terpenes have a wide range of biological properties, from antioxidant, anti-inflammatory, anti-cancer to neuroprotection (60), suggesting that terpenes might be expected to be developed as novel neuroprotective agents for patients with neurodegenerative diseases in the future. Additionally, the study showed that forest recreation lasting for 1 h and 45 min had a positive effect on the mental health of patients with affective disorders (61). The levels of four negative indicators of mood (nervous anxiety, depression, fatigue and confusion) measured by the POMS questionnaire were significantly decreased, and the vitality of positive mood index was significantly increased after the intervention. Tension-anxiety, depression-dejection, anger-hostility, and confusion were significantly reduced in patients with mental disorders. However, fatigue did not show a significantly change in response to forest bathing, suggesting that a symptom of schizophrenia (measured by fatigue) is likely to be difficult to change by forest therapy.

### 4.4 Sleep disorders

Insomnia is a prevalent sleep disorder that can be treated with drugs or non-drug therapies. Non-pharmacological treatments should be given priority due to the potential for medication addiction and side effects. Kim et al. conducted a 6-day forest bathing study on 35 postmenopausal women and found that forest bathing helped to improve sleep efficiency, reduce post-sleep awakenings, and increase total sleep time, which indicated that forest therapy can be used as a non-drug treatment (62). Furthermore, even though a short period of forest bathing for 2 days and 1 night, Kim et al. found that forest bathing only for 1 day could increase the salivary melatonin level and improve the sleep quality of medical staff who were working under high stress during the COVID-19 pandemic (63). Therefore, it is not difficult to infer that forest bathing might be a good alternative to nonpharmacological treatment for relieving insomnia in postmenopausal women.

## 5 Factors contributing to the health benefits of forest therapy

### 5.1 Tree species and forest density

The quantity and composition of phytocides produced by different tree species and plant organs varied. Isoprene was the main phytocide in deciduous forests, whereas a-pinene was the main phytocide in coniferous forests (64). The composition and content of phytochemicals in coniferous forests of different species were slightly different, with a-pinene being the main phytochemical in willow forests, followed by camphene; the amount of a-pinene was particularly high in Japanese Pinus sylvestris forests, followed by tricyclic terpenes, isoterpene pinene, limonene, etc., and the total amount of terpenes was higher than that in willow forests. Among the different plant organs, the content of phycidin released by leaves is higher, but the concentration of phycidin released by leaves of different tree species is different, which may be one of the main reasons for the different composition of phycidin in coniferous forests. Guan et al. (65) recruited 69 college students to visit an urban forest dominated by birch, maple and oak, and found that only academic anxiety was found to decrease in the maple forest, while the greatest reduction in employment stress anxiety was found in the birch forest. Students in the oak forest reported higher levels of academic anxiety than those in the birch forest. While in the oak forest, female participants experienced more anxiety relief than male participants. Sacchelli et al. (66) conducted a virtual reality study comparing four different forest types during the winter months and found that coniferous forests and Douglas firs were particularly good for stress relief. Moreover, high densities of trees threaten feelings of safety by limiting visual access and ease of movement, and may interfere with people's immersion in nature. On the contrary, forest environments with low vegetation densities can provide a sense of openness, but can also create a boring and monotonous landscape. Kim et al. (67) assessed the relationship between forest structural characteristics and treatment effects. The results showed that forest density altered treatment effects. Emotional and cognitive recovery showed the greatest effect in low forest density < 500/ha, and the therapeutic effect diminished as forest density increased.

### 5.2 Season and climate

Bach et al. analyzed monoterpene concentrations in a Holm's oak forest in the Mediterranean from June to November (68). The study found that total monoterpene concentrations has strong seasonal and daytime variability and peaks in summer. Specifically, the monthly mean diurnal cycle showed two distinct peaks in the morning (6:00–8:00) and afternoon (13:00–18:00) during July and August. This suggested that the seasonality of monoterpene concentrations is related to seasonal variations in plant monoterpene emissions, temperature, and other meteorological variables. Chen et al. studied the seasonal variation of terpenoid emission rate and composition in two species of cedar with different tree ages (69). A total of 21 terpenoids were detected, including 13 monoterpenoids, 4 sesquiterpenoids and 4 diterpenoids. Monoterpenes dominated emissions from both saplings and adult trees, producing more than 80% of terpene emissions. It was found that there were significant differences in the seasonal distribution of terpenoid emission rates between saplings and adult trees. Particularly, the actual total emissions of saplings were higher in the cold season than in the warm season, while the terpenoids emissions of adult trees were higher in the warm season. Bielinis et al. (70) investigated the effects of winter forest bathing on people in an environment where the ground and trees were covered with snow, and found that the negative mood index decreased significantly after exposure to the forest environment covered with snow and ice, while the positive “vitality” index did not increase or decrease significantly. Restorative mood increased significantly after forest sightseeing, but subjective vitality did not increase or decrease. Huang et al. explored the blood pressure lowering effect of forest bathing in camphor forest environment throughout the year and found that the blood pressure lowering effect of forest bathing in camphor forest environment changed with season (71). Except in winter, both diastolic blood pressure and systolic blood pressure decreased, while blood oxygen saturation increased after forest bathing. The effect of forest bathing on blood pressure reduction was obviously observed in summer and autumn. Zhou et al. (72) found that the composition and relative content of volatile organic compounds released by camphor trees varied between seasons, and the alkane volatile organic compounds varied greatly. The relative content of alkanes and organic acids in the VOCs released in spring and winter was higher than that of other VOCs, while the content of terpenes in the VOCs released in autumn and summer was higher. It was suggested that camphor forest bathing in spring, summer and autumn has a higher auxiliary regulation effect on blood pressure and inflammation in older adult patients with hypertension. Whereas, forest bathing in camphor forest in winter is not effective in the treatment of older adult patients with hypertension. The current study showed that the higher content of various volatile organic compounds in forests in spring, summer and autumn can produce better benefits for people's physiology and psychology when compared with those in winter.

### 5.3 Exposure environment

Due to various aspects such as work, the way and time of exposure to forests varies among urban populations, and different environmental and mode exposure may lead to different health outcomes. Song et al. have demonstrated that a 15-min walk in a well-managed artificial forest significantly reduced negative emotions-depression, anxiety, anger, fatigue, confusion and increased vitality when comparing to walking in an urban environment (73). Grazuleviciene et al. conducted an intervention study, in which volunteers were recruited to walk for 30 min in a city park or on a city street for seven days. A significant decrease in salivary cortisol levels was observed during the early phase of walking in a city park, whereas there was no significant effect on walking in the city streets (74).

Because of the medicinal effects of terpenes and terpenoids, it is believed that forest bathing has a positive effect on human health through exposure to forest aerosols containing these compounds, in addition to the physical relaxation effect. Terpenes are the largest class of natural organic compounds that may have anti-inflammatory, anti-cancer or neuroprotective effects (75). Monoterpenes belong to isoprenoids, which is the largest class of BVOCs and a major component of the forest atmosphere. Inhaling monoterpenes have been shown to reduce blood pressure and cortisol levels, improve the efficiency of antibiotic use (76) and boost the immune system (77, 78), particularly by increasing the percentage and activity of natural killer cells (79).

### 5.4 Exposure duration

The optimal time for forest exposure has long been debated, with researchers suggesting that even 15 min in the natural environment can have short-term beneficial effects on mental health, particularly coping with stress and anxiety (80). Meredith et al. have shown that sitting and/or walking in the forest for at least 10–30 min per day can reduce stress hormone levels, heart rate, blood pressure, and sympathetic nervous system activity and help improve mood and anxiety (81). Evidence from other studies suggests that the week spending in forest might be divided into different sessions (15 min twice a day for 3 consecutive days) and the beneficial effects of forest bathing should still be observed, both in terms of mood/self-reported health and in improving laboratory parameters of stress response and immune function (cortisol levels and NK cell counts) (82, 83). Notably, the beneficial effects of forest bathing on the immune system could persist for 7–30 days after the end of the forest bathing, which revealed that a monthly forest bathing might have some lasting benefits (84). Mao et al. demonstrated that participating in two 4-day forest-bathing trips, 4 weeks apart, had the added benefit of reducing the decline in brain natriuretic peptide levels, as well as alleviating inflammation and oxidative stress (85). Overall, based on a comprehensive review of the available evidences, forest bathing intervention for at least 2 hours per week could be thought of an excellent health-promoting activity, and if conditions do not permit, even once a month, in combination with other wholesome lifestyle habits, could be a beneficial practice to improve individual physical and mental health (86).

## 6 Conclusion and prospective

Forests are one of the main recreational destinations for urban populations. Evidences showed that forests and other natural environments are considered essential health resources that can play a role in disease prevention. Forest bathing makes use of various elements of the forest environment to help people maintain and improve their physical and mental health, coping with stress and promoting general health. As an alternative therapy for non-drug treatment of mental and psychological diseases, immersion in the forest environment through smelling, seeing, hearing, tasting and touching can regulate the body's nervous system, help relieve stress, alleviate depression, improve sleep quality of anxious people, enhance metabolic ability and immune function and promote population health.

Forest bathing has a positive effect on people's health. Exposure to the forest through a variety of activities can help people eliminate negative emotions, promote physical and mental health, and prevent physical and mental illnesses. With the acceleration of the pace of life, the increase in work stress and job burnout, and the impact of the COVID-19 pandemic, there is an unprecedented need to pay attention to life, health and development of forest healing program. Therefore, there is an urgent need to carry out more medical empirical research and clinical practice guidance programs on the effects of forest bathing on sub-health populations, such as the use of wearable sensor devices to monitor skin electrical activity, fingertip temperature, heart rate and blood pressure during forest bathing, and to assess the autonomic nervous system response to forest bathing, providing more evidences in clinical practice (87). Above all, countries, societies, organizations and individuals around the world need to take active action to raise awareness of the role of forest bathing in promoting people's health. Please do stop deforestation at global level.
